# Association between dysphagia risk and sleep quality in community-dwelling older adults: A cross-sectional study

**DOI:** 10.1016/j.heliyon.2024.e32028

**Published:** 2024-05-31

**Authors:** Yohko Hama, Sachiko Yamada, Rumi Nishimura, Mitsuyoshi Yoshida, Kazuhiro Tsuga, Emi Morita, Yudai Tamada, Yasufumi Kato, Yoko Kubo, Rieko Okada, Mako Nagayoshi, Takashi Tamura, Asahi Hishida, Kenji Wakai, Mariko Naito

**Affiliations:** a Department of Advanced Prosthodontics, Hiroshima University Graduate School of Biomedical and Health Sciences, Hiroshima, Japan; b Hiroshima Oral Health Center, Hiroshima, Japan; c Department of Oral Epidemiology, Hiroshima University Graduate School of Biomedical and Health Sciences, Hiroshima, Japan; d Department of Dentistry and Oral-Maxillofacial Surgery, Fujita Health University School of Medicine, Toyoake, Japan; e International Institute for Integrative Sleep Medicine, University of Tsukuba, Japan; f Forestry and Forest Products Research Institute, Forest Research and Management Organization, Japan; g Department of Preventive Medicine, Nagoya University Graduate School of Medicine, Nagoya, Aichi, Japan; h Department of International and Community Oral Health, Tohoku University Graduate School of Dentistry, Sendai, Miyagi, Japan

**Keywords:** Dysphagia risk, Sleep quality, PSQI-J, Community-dwelling older adults, Cross-sectional study

## Abstract

**Objectives:**

Exploring the effects of swallowing function on sleep quality could provide valuable insights into the potential impact of reduced swallowing function on sleep. However, pertinent studies are limited. Therefore, this study aimed to investigate the relationship between dysphagia risk and sleep health in community-dwelling older adults.

**Methods:**

Data for this cross-sectional study were obtained from the Shizuoka and Daiko studies conducted as part of the Japan Multi-Institutional Collaborative Cohort Study. Information on demographics, overall lifestyle, dysphagia risk, as well as sleep quality, duration, satisfaction, and regularity, was obtained using a self-administered questionnaire. Dysphagia risk and sleep quality were assessed using the Dysphagia Risk Assessment Questionnaire for the Community-dwelling Elderly and the Japanese version of the Pittsburgh Sleep Questionnaire Index, respectively. Multivariate logistic regression, adjusted for covariates, was employed to assess the association between dysphagia risk and sleep health.

**Results:**

Among the 3058 participants (1633 males, 1425 females) aged ≥60 years, 28.0 % exhibited dysphagia risk, and 19.1 % reported poor sleep quality. Those with dysphagia risk were more likely to experience poor sleep quality than those without dysphagia risk. In male participants, dysphagia was significantly associated with poor sleep quality, unsatisfactory sleep, and sleep irregularity, but was not significantly associated with unsatisfactory or irregular sleep in female participants. The Japanese version of the Pittsburgh Sleep Questionnaire Index components—subjective sleep quality, sleep latency, sleep disturbances, and daytime dysfunction—were associated with dysphagia risk in both sexes.

**Conclusions:**

Dysphagia risk is associated with sleep quality in older individuals in Japan. Thus, preserving swallowing function may contribute to enhancing sleep quality.

## 1 Introduction

Sleep disorders are common health concerns among older adults worldwide [1], with approximately 30 % of older adults in Japan experiencing such disorders [2]. Specifically, individuals aged 60 years and above tend to face increasing difficulties in maintaining sleep [3], potentially constituting a primary cause of sleep disorders in this demographic. Difficulty in maintaining sleep has been strongly associated with poor physical health conditions, including chronic pain, gastroenterological disorders, and respiratory disease [[4], [5], [6]]. Coughing has also been identified as a contributing factor [7]. In this regard, respiratory-swallowing coordination can also be impaired during sleep, leading to an increased occurrence of coughing after swallowing compared to that during wakefulness [8]. Healthy adults have been reported to typically exhibit lower amplitudes of pharyngeal swallowing pressure during sleep [9], increasing the likelihood of aspiration during sleep. Moreover, aging significantly reduces swallowing mechanisms and the motor sensation associated with swallowing function [10]. Dysphagia, a common problem among older individuals, affects approximately 30 % of community-dwelling older adults, nearly 50 % of geriatric patients, and over 50 % of nursing home residents [11], making it a prevalent issue in this demographic. As swallowing function declines with age, controlling swallowing during sleep may become increasingly difficult.

Although the effect of dysphagia on sleep quality remains largely unexplored, the potential influence of impaired swallowing function on sleep quality should be considered. Poor sleep quality has been associated with frailty [12], and poorer sleep quality and shorter sleep duration might be associated with Alzheimer's disease [13] in older populations. Furthermore, some older adults may be unaware of their poor sleep quality [14]. Objective assessments of sleep quality often require specialized modalities such as polysomnography and actigraphy [15], whereas decreased swallowing function can be observed using various screening methods [11]. In addition, swallowing function is expected to improve with rehabilitation. Understanding whether a relationship exists between maintaining swallowing function and preserving sleep quality could provide valuable insights into the potential impact of reduced swallowing function on sleep quality. Elucidating the relationship between dysphagia risk and sleep quality may highlight the importance of maintaining swallowing function and improving the health and well-being of older adults. It is anticipated that gaining a deeper understanding of the relationship between dysphagia risk and sleep quality may result in novel screening tools aimed at promoting safety and an improved quality-of-life for community-dwelling older adults. However, there is a notable absence of comprehensive studies that have thoroughly examined the relationship between dysphagia risk and sleep health on a large scale [12]. Therefore, in this study, we aimed to explore the association between dysphagia risk and sleep health in community-dwelling older adults and assess its relationship with sleep quality components.

## 2 Methods

### 2.1 Study participants

Data used in this cross-sectional study were obtained from the Shizuoka and Daiko studies conducted as part of the Japan Multi-Institutional Collaborative Cohort (J-MICC) Study [16]. The J-MICC study is a large cohort study designed to identify and evaluate gene-environment interactions within the context of lifestyle-related diseases. In the present study, we utilized data from participants in the second survey, conducted between February 2012 and March 2015. The Shizuoka study involved 5006 participants from individuals undergoing health checkups at the Seirei Preventive Health Care Center in Hamamatsu City, Shizuoka Prefecture, Japan [17], whereas the Daiko study enlisted 5152 participants from registered residents, mainly through posted leaflets, in Nagoya City [18]. A total of 10,158 individuals participated, and those with complete information on age, the Dysphagia Risk Assessment Questionnaire for the Community-dwelling Elderly (DRACE), and the Japanese version of the Pittsburgh Sleep Questionnaire Index (PSQI-J) were selected (n = 6437). Those younger than 60 years old (n = 3055), with missing data (n = 316), and who withdrew consent (n = 8) were excluded, resulting in a final cohort of 3058 participants for analyses.

All participants provided written informed consent after receiving a thorough explanation of the outline and objectives of the study. The study was approved by the Ethics Committee of the Nagoya University Graduate School of Medicine, Japan (approval numbers 1248 and 618) and the Ethical Committee for Epidemiological Research of Hiroshima University, Japan (approval numbers E2020-9253 and E202-9254). The study was performed in conformity with the Declaration of Helsinki and regulations in Japan. Analyses were performed using the dataset version 20190822.

### 2.2 Data collection and measurements

A self-administered questionnaire was distributed to all participants, which encompassed inquiries about various aspects, including sex, age, height, weight, mental health status, dysphagia risk, sleep health (quality, duration, satisfaction, and regularity), lifestyle factors, such as smoking and alcohol consumption status (never, former, or current), working status (worker or nonworker), disease history (cerebrovascular and cardiovascular disease), and regular use of sleep medication (more than once a week). Mental health status was assessed using the 12-item General Health Questionnaire (GHQ-12) [19], with a score ≥4 indicating poor mental health. All data were reviewed by trained staff.

### 2.3 Dysphagia risk assessment

Dysphagia risk was assessed using the DRACE [20]. The DRACE was specifically designed to identify potential swallowing-related issues among frail older adults residing in the community. It consists of 12 items, each assigned a score ranging from 0 to 2, resulting in a cumulative score that spans from 0 to 24. A total DRACE score of ≥4 indicated an elevated risk of dysphagia [21], and this cutoff value was used in our study.

### 2.4 Sleep health assessment

Sleep health was assessed across four dimensions: quality, duration, satisfaction, and regularity [22]. Sleep quality was assessed using the PSQI-J [23,24]. The PSQI-J encompasses 19 items that include seven components: subjective sleep quality, sleep latency, sleep duration, habitual sleep efficiency, sleep disturbance, use of sleep medication, and daytime dysfunction. Each component is rated on a four -point scale (0–3), resulting in a global PSQI score ranging from 0 to 21, with a higher PSQI score indicating worse sleep quality. A score ≥6 in the PSQI-J indicated poor sleep quality. We also assessed the PSQI-J components, score 0 vs. score 1–3. Habitual average sleep duration was assessed by asking the following questions, in addition to the PSQI-J question items: “How many hours do you usually sleep?” Regarding sleep satisfaction, participants were asked, “Do you think you get enough sleep?” Responses to the questions were categorized as 1: enough; 2: somewhat not enough; 3: not enough; and 4: I do not know. Finally, we inquired about sleep regularity with the following questions: “Are your bedtimes and wake times regular?” to which participants could respond as either “regular” or “irregular.”

### 2.5 Statistical analysis

The mean and standard deviation values are presented, and t-tests were utilized to compare continuous data between the groups without (DRACE <4) and with (DRACE ≥4) dysphagia risk. Categorical data were tabulated using frequencies and percentages, and the chi-square test was employed to compare between group differences. Multivariate logistic regression analysis was performed to identify the association between dysphagia risk and sleep health parameters (quality, duration, satisfaction, and regularity) based on three models. Model 1 was adjusted for age and research site. Model 2 incorporated the adjustments from Model 1, along with GHQ-12, body mass index (BMI), cerebrovascular and cardiovascular disease, as well as smoking, alcohol consumption, and working statuses. Model 3 was adjusted as in Model 2 while excluding participants taking sleep medication or those with insomnia or sleep apnea syndrome (SAS). Similarly, we assessed the association between dysphagia risk and PSQI-J components, adjusted in Model 3. Sleep duration was categorized as less than 6 h (short sleep duration) and 6 h or more. Sleep satisfaction was categorized as “enough” or other responses. Mean and standard error values for PSQI-J component scores (subjective sleep quality, sleep latency, sleep duration, habitual sleep efficiency, sleep disturbance, daytime dysfunction) were obtained using analysis of covariance for comparisons between groups with and without dysphagia risk, with adjustments as specified in Model 3. One of the PSQI-J components, “use of sleep medication,” was excluded owing to the exclusion of participants taking sleep medication in Model 3.

All calculations and statistical tests were performed using the SPSS software (version 28 for Windows; IBM, Armonk, NY). Results are presented as adjusted odds ratios (ORs) with 95 % confidence intervals (95 % CI). P-values <0.05 were considered statistically significant.

## 3 Results

### 3.1 Characteristics of the study participants

Among the 3058 participants (1633 males, 1425 females; mean age, 66.5 ± 4.2 years), 28.0 % exhibited dysphagia risk, whereas 19.1 % experienced poor sleep quality. Compared to those without dysphagia risk, individuals at risk of dysphagia were more likely to experience poor sleep quality (28.5 % vs. 15.4 %, P < 0.001) and had significantly shorter average sleep time (6.74 ± 0.88 vs. 6.62 ± 0.99, P = 0.003). Further, fewer individuals at risk of dysphagia answered that they had sufficient sleep (61.7 % vs. 48.9 %, P < 0.001), and many had irregular sleep patterns (7.3 % vs. 13.7 %, P < 0.001) (Table 1, Supplement 1) when compared to those without dysphagia risk. Table 1 shows the characteristics of the study population according to dysphagia risk by sex. Age, proportion of poor sleep quality, unsatisfactory sleep, and irregular sleep were significantly higher in the dysphagia risk group in both sexes. A shorter average sleep duration was observed in female participants in the dysphagia risk group. No difference in BMI was observed between male and female participants with or without dysphagia risk.

**Table 1:** Characteristic of the study population according to dysphagia risk by sex.

		Male participants					Female participants				
		Without dysphagia risk		With dysphagia risk		*P*	Without dysphagia risk		With dysphagia risk		*P*
		n	Weight％	n	Weight％		n	Weight％	n	Weight％	
N		1208	74.0	425	26.0		995	69.8	430	30.2	
**Age, mean ± SD**		66.30 ± 4.14		67.11 ± 4.17		＜0.001	66.23 ± 4.15		67.32 ± 4.21		＜0.001
**Sleep quality**											
Good		1071	88.4	314	73.9	＜0.001	796	80.0	297	69.1	＜0.001
Poor		314	11.6	111	26.1		199	20.0	133	30.9	
**Sleep duration, mean ± SD**		6.85 ± 0.86		6.78 ± 1.01		0.168	6.61 ± 0.89		6.47 ± 0.96		0.012
<6 h		88	7.3	47	11.1	0.042	112	11.3	73	17.0	0.009
6–8 h		829	68.6	273	64.2		744	74.8	294	68.4	
>8 h		291	24.1	105	24.7		138	13.9	63	14.6	
**Sleep satisfaction**											
Enough		815	67.7	221	52.0	＜0.001	540	54.5	197	45.8	0.002
Somewhat enough		334	27.7	164	38.6		347	35.0	160	37.3	
Not enough		40	3.3	30	7.1		58	5.8	44	10.2	
Neither		16	1.3	10	2.4		47	4.7	29	6.7	
**Sleep regularity**											
Regular		1110	94.3	364	87.3	＜0.001	890	90.8	362	85.4	0.003
Irregularity		67	5.7	53	12.7		90	9.2	62	14.6	
***GHQ-12***											
Score＜4		1075	89.0	332	78.1	＜0.001	809	81.3	264	61.4	＜0.001
≥4		133	11.0	93	21.9		186	18.7	166	38.6	
**BMI, mean ± SD**		23.06 ± 2.54		22.92 ± 2.68		0.339	21.82 ± 2.84		21.76 ± 2.67		0.689
**Smoking status**											
Current		128	10.6	41	9.6	0.760	22	2.2	18	4.2	0.086
Former		688	56.9	250	58.8		71	7.1	35	8.1	
Never		392	32.5	134	31.5		901	90.7	376	87.7	
**Alcohol drinking status**											
Current		886	73.3	307	72.2	＜0.001	331	33.3	154	35.8	0.647
Former		25	2.1	24	5.6		11	1.1	5	1.2	
Never		297	24.6	94	22.1		652	65.6	271	63.0	
**Working status**											
Worker		667	55.6	201	47.3	0.003	375	37.8	129	30.0	0.005
Non worker		532	44.4	224	52.7		617	62.2	3031	70.0	
**Cerebrovascular disease**											
Yes		13	1.1	5	1.2	0.870	12	1.2	5	1.2	0.939
No		1188	98.9	419	98.8		979	98.8	425	98.8	
**Cardiovascular disease**											
Yes		71	5.9	30	7.1	0.391	28	2.8	18	4.2	0.183
No		1131	94.1	394	92.9		963	97.2	412	95.8	
**Taking sleep medication**											
Yes		49	4.1	56	13.3	＜0.001	85	8.6	78	18.1	＜0.001
No		1157	95.9	366	86.7		909	91.4	352	81.9	
**Insomnia**											
Yes		19	1.6	27	6.4	＜0.001	32	3.2	33	7.7	＜0.001
No		1180	98.4	394	93.6		957	96.8	394	92.3	
**Sleep apnea syndrome**											
Yes		52	4.3	24	6.4	0.171	17	1.7	10	2.3	0.492
No		1145	95.7	395	94.3		970	98.3	420	97.7	

BMI, body mass index; SD, standard deviation; GHQ-12, 12-item General Health Questionnaire.

### 3.2 Association between dysphagia risk and sleep health

The results of the multivariate-adjusted ORs of sleep quality, duration, satisfaction, and regularity according to dysphagia risk are shown in Table 2. Among male participants, those with dysphagia risk exhibited a significant tendency toward experiencing poor sleep quality (OR, 2.0; 95 % CI, 1.4–2.8), unsatisfactory sleep (OR, 1.7; 95 % CI, 1.3–2.2), and sleep irregularity (OR, 1.9; 95 % CI, 1.2–3.0). Among female participants, those with dysphagia risk exhibited a significant tendency toward experiencing poor sleep quality (OR, 1.4; 95 % CI, 1.0–2.0) and short sleep duration (OR, 1.5; 95 % CI, 1.0–2.1). However, the association was not statistically significant for unsatisfactory or irregular sleep.

**Table 2:** Multivariate-adjusted odds ratios for sleep quality, sleep duration, sleep satisfaction, and sleep regularity based on risk of dysphagia.

	Dysphagia risk	No.	Model 1			Model 2			No.	Model 3		
			OR	(95 % CI)	*P*	OR	(95 % CI)	*P*		OR	(95 % CI)	*P*
**Sleep quality**		Poor/good							Poor/good			
Male participants	Without	140/1068	1	(Reference)		1	(Reference)		102/995	1	(Reference)	
	With	111/314	2.616	(1.973–3.470)	＜0.001	2.303	(1.716–3.091)	＜0.001	63/279	1.980	(1.384–2.832)	<0.001
Female participants	Without	199/796	1	(Reference)		1	(Reference)		138/735	1	(Reference)	
	With	133/297	1.684	(1.298–2.186)	＜0.001	1.447	(1.113–1.899)	0.008	80/260	1.385	(1.001–1.917)	0.049
**Sleep duration**		<6 h/≥ 6 h							<6 h/≥ 6 h			
Male participants	Without	88/1120	1	(Reference)		1	(Reference)		73/1024	1	(Reference)	
	With	47/378	1.624	(1.113–2.371)	0.012	1.604	(1.086–2.370)	0.018	34/308	1.533	(0.998–2.377)	0.056
Female participants	Without	112/882	1	(Reference)		1	(Reference)		97/776	1	(Reference)	
	With	73/357	1.576	(1.139–2.178)	0.006	1.483	(1.061–2.075)	0.021	55/285	1.474	(1.015–2.141)	0.042
**Sleep satisfaction**		Other/sufficient							Other/sufficient			
Male participants	Without	374/815	1	(Reference)		1	(Reference)		325/755	1	(Reference)	
	With	194/221	1.899	(1.504–2.397)	＜0.001	1.791	(1.406–2.281)	＜0.001	142/192	1.685	(1.293–2.195)	<0.001
Female participants	Without	405/540	1	(Reference)		1	(Reference)		344/488	1	(Reference)	
	With	204/197	1.372	(1.081–1.741)	0.009	1.187	(0.926–1.523)	0.177	151/168	1.131	(0.860–1.486)	0.378
**Sleep regularity**		Irregularity/regular							Irregularity/regular			
Male participants	Without	67/1110	1	(Reference)		1	(Reference)		59/1011	1	(Reference)	
	With	54/364	2.395	(1.628–3.525)	＜0.001	2.090	(1.400–3.118)	＜0.001	37/299	1.879	(1.201–2.939)	0.006
Female participants	Without	90/890	1	(Reference)		1	(Reference)		76/784	1	(Reference)	
	With	62/362	1.582	(1.114–2.246)	0.010	1.395	(0.967–2.011)	0.075	42/292	1.212	(0.796–1.845)	0.369

BMI, body mass index; CI, confidence interval; GHQ-12, the 12-item General Health Questionnaire; OR, odds ratios.

Model 1: Adjusted for age and research site.

Model 2: Adjusted for Model 1, plus GHQ-12, BMI, cerebrovascular disease, cardiovascular disease, smoking, alcohol consumption, and working status.

Model 3: Same as in Model 2, with participants taking sleep medication, or with insomnia or sleep apnea syndrome excluded.

### 3.3 PSQI-J component scores between groups with and without dysphagia risk

**Fig. 1:**
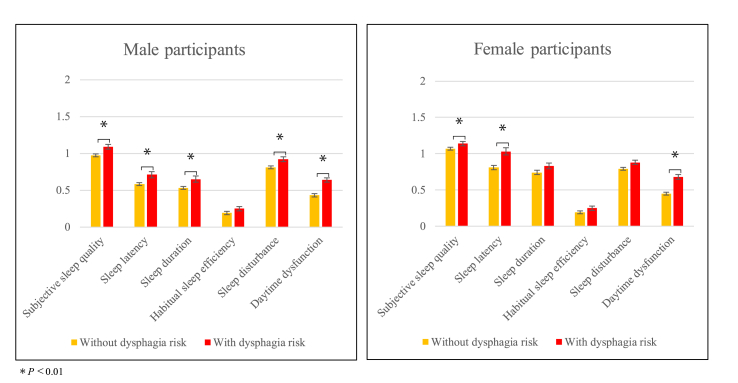
Comparisons of PSQI-J component scores between groups without and with dysphagia risk. The components were adjusted for age, research site, GHQ-12, BMI, cerebrovascular disease, cardiovascular disease, smoking, alcohol consumption, and working status, after exclusion of participants taking sleep medication, or with insomnia or sleep apnea syndrome. BMI, body mass index; GHQ-12, the 12-item General Health Questionnaire; PSQI-J, the Japanese version of the Pittsburgh Sleep Questionnaire Index.

Fig. 1 shows the significant differences between groups with and without dysphagia risk. The means of subjective sleep quality, sleep latency, and daytime dysfunction scores were significantly higher in both male and female participants with dysphagia risk than in those without (P < 0.01).

The results of the logistic regression analysis of the associations between dysphagia risk and the PSQI-J components are shown in Table 3. A correlation was observed between dysphagia risk and subjective sleep quality, sleep latency, sleep disturbance, and daytime dysfunction in male and female participants. Specifically, the OR of dysphagia risk between those with daytime dysfunction score of 1–3 was 2.19 in male and 2.04 in female participants, in contrast to those whose score was 0.

**Table 3:** Multivariate-adjusted odds ratios for PSQI-J components according to dysphagia risk.

	Dysphagia risk		Male participants				Female participants		
		N	OR	95 % CI	*P*	N	OR	95 % CI	*P*
		Score 0/1-3				Score 0/1-3			
Subjective sleep quality	Without	100/909	1	(Reference)		124/749	1	(Reference)	
	With	37/305	1.587	(1.083–2.327)	0.018	24/316	1.860	(1.164–2.971)	0.009
Sleep latency	Without	621/476	1	(Reference)		373/500	1	(Reference)	
	With	154/188	1.463	(1.139–1.878)	0.003	100/240	1.563	(1.180–2.070)	0.002
Sleep duration	Without	634/463	1	(Reference)		391/482	1	(Reference)	
	With	183/159	1.200	(0.932–1.544)	0.656	138/202	1.166	(0.895–1.520)	0.255
Habitual sleep efficiency	Without	942/155	1	(Reference)		757/116	1	(Reference)	
	With	277/65	1.298	(0.932–1.807)	0.123	276/64	1.334	(0.941–1.891)	0.106
Sleep disturbance	Without	264/833	1	(Reference)		233/640	1	(Reference)	
	With	55/287	1.528	(1.102–2.120)	0.011	61/279	1.392	(1.004–1.931)	0.047
Daytime dysfunction	Without	692/405	1	(Reference)		543/330	1	(Reference)	
	With	151/191	2.095	(1.619–2.711)	<0.001	136/204	2.035	(1.396–2.458)	<0.001

BMI, body mass index; CI, confidence interval; GHQ-12, the 12-item General Health Questionnaire; OR, odds ratios; PSQI-J, the Japanese version of the Pittsburgh Sleep Questionnaire Index.

Adjusted for age, research site, GHQ-12, BMI, cerebrovascular disease, cardiovascular disease, smoking, alcohol consumption, and working status, after exclusion of participants taking sleep medication, or with insomnia or sleep apnea syndrome.

## 4 Discussion

In this study, we investigated the association between dysphagia risk and sleep quality, duration, satisfaction, and regularity in community-dwelling older adults aged ≥60 years. The main results show that dysphagia risk was associated with poor sleep quality and sleep deprivation in both male and female participants, and male participants were more likely to experience lower sleep satisfaction and irregular sleep. Regarding the association between dysphagia risk and PSQI components, dysphagia risk was associated with subjective sleep quality, sleep latency, and daytime dysfunction. Subjective sleep quality was related to sleep efficiency, with poor subjective sleep quality being associated with sleep deprivation, poor sleep efficiency, and frequent nocturnal awakenings [25]. Sleep efficiency was not associated with dysphagia risk; therefore, the risk of dysphagia was more strongly associated with difficulty in maintaining sleep owing to frequent nocturnal awakenings.

Sleep-disordered breathing (SDB) may be a cause of difficulty in maintaining sleep owing to dysphagia risk. SDB encompasses conditions that cause abnormal breathing during sleep, with SAS being the most common. SDB increases with age, with other contributing factors, such as decreased compliance of the upper airway muscles, increased collapsibility, changes in respiratory chemoreceptor sensitivity, and destabilization of sleep architecture leading to altered respiratory thresholds [26]. This can result in shallow breathing and episodes of unconscious breathlessness throughout the night, ultimately causing difficulty in maintaining sleep. In this study, only a few patients had a history of SAS. SAS tends to be more prevalent in older adults with low obesity rates, minimal nocturnal oxygen desaturation, and few symptoms of daytime sleepiness [26]. Therefore, some cases of SAS might have been underdiagnosed.

Dry mouth is also a potential cause of difficulty in maintaining sleep, as it has been reported to decrease sleep quality. Dry mouth is more prevalent among the older population [27] and can lead to difficulty in swallowing [28], which may contribute to reduced sleep quality [29].

Pathological conditions that induce coughing during sleep, such as aspiration and acid reflux, can be considered potential causes. Aging, along with reduced salivary gland function during sleep, decreases clearance in the throat and esophagus, thereby weakening the defense mechanisms of the airway [30]. Gastroesophageal reflux affects sleep disturbances [31], and nighttime acid reflux is associated with awakenings during sleep [32]. When stomach acid stagnant in the throat is aspirated into the airway, it can trigger chemical inflammation and induce coughing. While saliva helps neutralize the acid in the throat and esophagus during the day, reduced saliva production during sleep, along with the supine position, promotes gastroesophageal reflux, exacerbating this condition and leading to frequent interruptions during nighttime sleep. Although clinical data on gastroesophageal reflux were not collected in the present study, daytime dysfunction was associated with dysphagia risk. Previous research has demonstrated a significant correlation between daytime sleepiness and swallowing disorders [33], with gastroesophageal reflux during sleep as one of the contributing factors [34]. In the present study, the presence of swallowing disorder risk was associated with approximately twice the risk of daytime sleepiness. Therefore, sleep interruptions caused by gastroesophageal reflux may impair sleep quality and exacerbate daytime sleepiness.

The results of this study indicate that men at risk of dysphagia experience reduced sleep satisfaction and a tendency toward irregular sleep. Frequent sleep interruptions have been reported to lower sleep satisfaction [35], and sleep regularity is influenced by factors such as lifestyle, circadian rhythms, and daily schedules [22]. Therefore, difficulty in maintaining sleep can also affect sleep satisfaction and regularity. These associations observed in men may be attributed to a higher risk of SAS [36]. In the present study, the percentage of male participants in the dysphagia risk group who were employed was 47.3 %, higher than that of female participants, which was 37.8 %. Furthermore, the percentage of male participants employed full-time was 60 %, whereas that of female participants was less than 30 %. This trend suggests that men may have longer working hours, leading to irregular sleep patterns. Moreover, age-related decreases in deep, non-rapid eye movement sleep, are observed only in men [37], which could contribute to shallower sleep patterns in men. The results of the analysis of the differences in PSQI-J scores revealed that male participants had greater variations in scores on several items, suggesting that men tend to experience shallower sleep than women. The observed sex differences might be due to differences in lifestyle, in addition to the differences in skeletal structure and lifestyle patterns. Compared to female participants, male participants were approximately four times more likely to have a current smoking status and approximately 2.5 times more likely to have an alcohol drinking status. We propose that these lifestyle habits may also affect sleep health.

In this study, the presence of dysphagia risk in older individuals led to poor sleep quality. Poor sleep quality in older individuals is not only associated with cognitive impairment [38] but also with deterioration in self-rated health status [39], decreased physical capabilities [40], and frailty [12]. Dysphagia is also significantly correlated with skeletal muscle strength in community-dwelling older individuals [41]. Thus, the worsening of skeletal muscle strength may be further exacerbated when sleep quality declines owing to swallowing disorders. Moreover, prolonged daytime sleepiness has been linked to an increased risk of falls, sarcopenia [42], and cognitive decline [43]. Dysphagia is also related to sarcopenia, and swallowing function is significantly reduced in older individuals with sarcopenia even before the clinical symptoms become apparent [44]. While the community-dwelling older participants in the present study were relatively healthy, there is a potential for a vicious cycle of declining physical function owing to the presence of swallowing disorders and subsequent decline in sleep quality. This underscores the significance of considering dysphagia risk and sleep health as interconnected factors.

Unhealthy sleep can predominantly manifest as insufficient sleep, affecting three primary dimensions: sleep duration, reduced sleep satisfaction, and poor sleep efficiency [45]. The risk of dysphagia may influence all of these dimensions. While healthy older individuals often compensate for quantitative changes in nighttime sleep by increasing daytime naps without a noticeable drop in sleep satisfaction [46], they may not readily recognize a decline in sleep quality. However, a deterioration in the swallowing function is a noticeable symptom that can be easily identified by both the individuals and observers. Dysphagia leads to reduced sleep quality, which may further exacerbate physical frailty and create a vicious cycle. Activities, such as physical exercise, oral health instruction, and nutritional guidance, may contribute to the improvement or maintenance of swallowing function in older individuals [47]. Maintaining swallowing function can be considered as a motivation to preserve sleep quality and overall health. Improving sleep quality could positively impact both life expectancy and overall well-being, although further studies are required to substantiate our findings. This study sheds light on a previously understudied area, unveiling a significant association between dysphagia risk and sleep quality in older adults. This discovery underscores the significance of preserving optimal swallowing function as a potential means to improve sleep quality, providing valuable insights for clinical interventions aimed at enhancing the overall well-being of this population.

This study had some limitations. First, it was a cross-sectional study, which raises the possibility of reverse causality. It is also possible that poor sleep health may increase the risk of dysphagia. Thus, a long-term longitudinal study is essential to determine the extent to which maintaining swallowing function may impact sleep health. Second, the data on dysphagia risk and sleep health measures were self-reported. Questionnaire surveys have been reported to underestimate the prevalence of swallowing disorders [11]. However, the estimated prevalence of swallowing disorders in community-dwelling older adults is approximately 30 % [11], consistent with the proportion of swallowing disorder risk in this study, as determined using DRACE. Clinical evaluation is required for a definitive diagnosis of dysphagia. However, objective assessment measures of dysphagia, such as video fluoroscopy and fiberoptic endoscopic evaluation of swallowing, and swallowing assessments for sleep quality, such as polysomnography and actigraphy, are unsuitable for large-scale surveys. PSQI is a frequently used subjective measurement of sleep quality, with good internal reliability and validity [48]; however, defining and objectively measuring sleep quality can be difficult, considering the significant variability among individuals and its subjective nature [48]. The validity of the information concerning sleep duration, satisfaction, and regularity, which was acquired using subjective questionnaires, has not been established; however, we consider that their versatility, including their many potential interpretations, makes them suitable for application in future epidemiological studies. Third, the data in this study were obtained from individuals undergoing health checkups or participating in postings, potentially resulting in a healthier sample than the general community-dwelling older population. Despite these limitations, an association was observed between the risk of dysphagia and sleep health. Therefore, further investigations in different populations are important. In addition, this study was conducted in a Japanese population, which potentially limits the generalizability of the findings. It remains unclear whether similar results would be observed in different ethnic groups or among other cultures. Fourth, we were unable to investigate the disease history of gastroesophageal reflux disease or dry mouth, which may cause difficulty maintaining sleep, as we did not have data, and as stated previously, clinical evaluation for dysphagia is unsuitable for a large-scale survey. Further research is required based on these data.

## 5 Conclusions

In this study, the risk of dysphagia was associated with sleep quality in older individuals in Japan. Strategies focused on maintaining swallowing function, including physical exercise, oral health instructions, and nutritional guidance, may contribute to the improvement of sleep quality. However, further studies are required to validate our findings.

## Ethical Statement

The study was approved by the Ethics Committee of the Nagoya University Graduate School of Medicine, Japan (approval numbers 1248 and 618) and the Ethical Committee for Epidemiological Research of Hiroshima University, Japan (approval numbers E2020-9253 and E2020-9254). The study was performed in conformity with the Declaration of Helsinki and regulations in Japan. Analyses were performed using the dataset version 20190822. All participants provided written informed consent after receiving a thorough explanation of the outline and objectives of the study.

## Funding

This study was funded by Grants-in-Aid for Scientific Research (KAKENHI) on Priority Areas of Cancer (No.17015018), Innovative Areas (No.221S0001), and Grants-in-Aid for Scientific Research (KAKENHI) (No.JP16H06277) from the 10.13039/501100001700Ministry of Education, Culture, Sports, Science and Technology, Japan. The funder had no role in the design of the study, collection, analysis, data interpretation, or manuscript writing.

## Data availability statement

The data that support the findings of this study are available from the corresponding author upon reasonable request.

## CRediT authorship contribution statement

**Yohko Hama:** Writing – review & editing, Writing – original draft, Visualization, Investigation, Formal analysis, Data curation, Conceptualization. **Sachiko Yamada:** Data curation. **Rumi Nishimura:** Data curation. **Mitsuyoshi Yoshida:** Writing – review & editing, Supervision. **Kazuhiro Tsuga:** Writing – review & editing, Supervision. **Emi Morita:** Writing – review & editing, Supervision. **Yudai Tamada:** Writing – review & editing, Supervision. **Yasufumi Kato:** Data curation. **Yoko Kubo:** Writing – review & editing, Data curation. **Rieko Okada:** Data curation. **Mako Nagayoshi:** Data curation. **Takashi Tamura:** Writing – review & editing, Data curation. **Asahi Hishida:** Data curation. **Kenji Wakai:** Writing – review & editing, Visualization, Funding acquisition. **Mariko Naito:** Writing – review & editing, Validation, Funding acquisition, Formal analysis, Conceptualization.

## Declaration of competing interest

The authors declare that they have no known competing financial interests or personal relationships that could have appeared to influence the work reported in this paper.
