# Supplementary material for: Association between dysphagia risk and sleep quality in community-dwelling older adults: A cross-sectional study
Source: Heliyon. 2024 May 31;10(11):e32028. doi: 10.1016/j.heliyon.2024.e32028 (PMC11180309; doi:10.1016/j.heliyon.2024.e32028)
Supplement: Supplementary file 1 [file mmc1.docx]

Supplement1

Characteristic of the study population by dysphagia

	Total	
	DRACE＜4	
	n	Weight%
N	2203	72.0
**Age, mean±SD**	66.27±4.15	
**Sleep quality**		
Good	1864	84.6
Poor	339	15.4
**Sleep duration, mean±SD**	6.74±0.88	
< 6 h	200	9.1
6-8 h	1573	71.4
> 8 h	429	19.5
**Sleep satisfaction**		
Enough	1355	61.7
Somewhat enough	681	31.0
Not enough	98	4.4
Neither	63	2.9
**Sleep regularity**		
Regular	2000	92.7
Irregularity	157	7.3
GHQ-12		
Score＜4	1884	85.5
≥ 4	319	14.5
**BMI, mean±SD**	22.50±2.75	
**Smoking status**		
Current	150	6.8
Former	759	34.5
Never	1293	58.7
**Alcohol drinking status**		
Current	1217	55.3
Former	36	1.6
Never	949	43.1
**Working status**		
Worker	1042	47.6
Non worker	1149	52.4
**Cerebrovascular disease**		
Yes	25	1.1
No	2167	98.9
